# Mango Firmness Modeling as Affected by Transport and Ethylene Treatments

**DOI:** 10.3389/fpls.2018.01647

**Published:** 2018-11-20

**Authors:** Rob E. Schouten, Shuang Fan, Julian C. Verdonk, Yuchen Wang, Nur Fauzana Mohd Kasim, Ernst J. Woltering, L. M. M. Tijskens

**Affiliations:** ^1^Horticulture and Product Physiology, Wageningen University, Wageningen, Netherlands; ^2^Wageningen Food and Biobased Research, Wageningen, Netherlands

**Keywords:** kinetic modeling, softening, sourcing, sources of variation, mango, firmness, transport simulation

## Abstract

More and more, tropical fruit are subjected to accelerated ripening at receiving markets until “ready to eat.” We propose a kinetic model that incorporates the effects of temperature and ethylene on the firmness behavior of “Keitt” and “Kent” mangoes. Stiffness of individual mangoes, as measured by the acoustic firmness tester, was measured repeatedly over time. The firmness model assumes fixed levels of ethylene, established after the climacteric peak, that steadily induces production of softening enzymes that subsequently denaturalize. The initial level of these enzymes is assumed to be zero due to either the tree factor for freshly harvested mangoes, or due to chilling injury for reefer transported mangoes. The kinetic parameter set for “Keitt” mangoes was estimated based on a Spanish batch, freshly harvested and ripened under dynamic temperature scenarios, combined with a reefer transported Brazilian batch stored at four constant temperatures. Firmness data from reefer transported batches, from Brazil, Ivory Coast and Mali, stored at four constant temperatures were used to estimate a set of kinetic parameters for the “Kent” mangoes. Only a partial set of “Kent” kinetic parameters could be established due to the often already advanced stage of softening at the time of arrival. The effect of ethylene was investigated by applying external ethylene levels, varying from 0 to 100 μL L^−1^. The effect of external application of ethylene was modeled by estimating EF, the ethylene factor, being a reflection of the internal ethylene level and ethylene sensitivity. The effect of ethylene application on softening was sometimes huge. For an Israeli “Keitt” batch a fifty times higher EF was found when the firmness behavior of low- (without ethylene application) and high temperature (with ethylene application) stored sub-batches were compared. However, this effect was sometimes also small, especially for reefer transported mangoes. For commercial application, a reliable prediction of the time until “ready to eat” is not possible because of the current inability to assess EF. Nevertheless, the proposed model described mango softening accurately, irrespective of the sourcing area and includes the effects of storage temperature and ethylene application.

## Introduction

The European demand for mangoes (*Mangifera indica* L.,) shows an ever-increasing trend with total EU imports worth around €402 million in 2014 (CBI Market Intelligence, 2015). Most of these mangoes have been transported for several weeks in refrigerated containers. Mango losses in the postharvest chain are high due to a number of reasons, e.g., improper maturity at harvesting, mechanical damage, sap burn, spongy tissue, lenticels discoloration, chilling injury, and disease incidence (Sivakumar et al., 2011). In Europe, the leading importing country for fresh mangoes is the Netherlands. Interestingly, the Netherlands is also the leading mango export country, re-exporting more than 75% in 2013 (FAO, 2016). In the Netherlands, most of the imported mangoes will be ripened additionally before supplying them to the market. To provide customers with “ready to eat” (RTE) tropical fruit, ripening protocols are becoming increasingly important to the retail sector. The RTE concept aims to guarantee that fruit are soft enough to be eaten immediately or within a few days after purchase. Most of the mango ripening protocols are adaptations of existing banana ripening protocols. These protocols have been steadily improved over the years based on experience, by modifying storage time and temperature, to take cultivar, sourcing area and harvest season into account.

Softening of mango occurs through enzyme-mediated alteration in the structure and composition of cell wall, partial or complete solubilisation of cell wall polysaccharides, and hydrolysis of starch and other polysaccharides (Fuchs et al., 1980). Cell wall degrading enzymes such as polygalacturonase, (both exo- and endo- forms), pectin lyase, endo-1,4-β-D-glucanase (Zaharah et al., 2013) and galactanase, arabinanase and β-galactosidase (Prasanna et al., 2003) showed increased activity linked to ethylene biosynthesis. However, the activity of pectin esterase (Roe and Bruemmer, 1981) and pectin methyl esterase (Prasanna et al., 2003) decreases after the climacteric peak. For mango, also less well-known cell wall degrading enzymes play a role in softening, such as endo-mannanase, α-mannosidase (Yashoda et al., 2007) and an early ethylene responsive α-expansin (Sane et al., 2005). Likely, abscisic acid triggers the climacteric in mango by inducing ethylene biosynthesis enzymes and accumulation of the direct ethylene precursor and subsequent production of cell wall degrading enzymes (Zaharah et al., 2013).

The pivotal role of ethylene in mango softening is recognized. Mangoes exposed to exogenous ethylene levels ranging from, 0.005 to 10 μL L^−1^ at 20°C, showed a decrease in time to ripen from 12.8 to 7.5 days (Wills et al., 2001). Adding 100 μL L^−1^ ethylene for 12 h at 25°C reduced the ripening time of “Ataulfo” mangoes by 4 days (Montalvo et al., 2007). Fruit treated with the ethylene inhibitor 1-MCP maintained firmness while fruit treated with 5 μL L^−1^ exogenous ethylene resulted in increased softening (Wang et al., 2009). In fact, 100 μL L^−1^ ethylene is recommended for accelerated and uniform ripening (Kader, 1997). However, in commercial mango ripening facilities in the Netherlands the use of exogenous ethylene is more and more abandoned as softening often seems hardly affected. Literature on the effect of ethylene on ripening is mostly based on experiments with freshly harvested mangoes, and not with reefer transported mangoes. Mango softening models are rare. De Ketelaere et al. (2006) proposed a logistic model to describe mango softening including the quantification of the biological and technical variation. This model, however, does not incorporate the effect of ethylene. A recent kiwi modeling paper focused on softening for a range of temperatures and exogenous ethylene levels affecting the breakdown of cell wall compounds by an autocatalytic enzyme system that is activated by ethylene (Hertog et al., 2016).

The aim of this research is to develop a kinetic model, based on simplified physiological concepts, that describes softening of mango as affected by temperature, cultivar, sourcing region, and ethylene application. We describe model development, estimation of the model parameters for both “Keitt” and “Kent” mangoes, transferability of the kinetic parameters and describe the sources of variation. Finally, the factors that currently limit the applicability of the model are discussed as to enable the ripening industry to start with applying science based ripening protocols for RTE mangoes.

## Materials and methods

### Firmness measurements

Non-destructive stiffness was measured using a commercial acoustic firmness tester (AFS, AWETA, Nootdorp, the Netherlands) with the tick power of the plunger set to 16. The AFS combines a resonant frequency (*f* in Hz) and mass (*m*, in kg), measured by an inbuild balance, into a *FI* (firmness index) (Schotte et al., 1999) expressed according to Equation 1.

(1) $$\begin{array}{l} FI=\frac{f^{2}m^{2/3}}{10^{4}} \end{array}$$

For spherical fruit without a clear internal structure, the resonance frequency spectrum will be dominated by one large peak. However, for ellipsoid stone fruit like mango the resonance spectrum will be more complex with at least two dominating peaks. Cherng and Ouyang (2003) derived a stiffness index on a theoretical basis for ellipsoid fruit that includes two resonant frequencies of the lowest spherical modes. Initial results showed that for mango both peaks move together to a lower frequency during storage and merge to one peak when the fruit is soft. Since both peaks that determine stiffness coincide when the fruit is soft, it was decided that one frequency would be sufficient to characterize stiffness. The peak at higher frequency shifts over a larger frequency range during softening and has thus a higher measurement sensitivity. Therefore, only the frequency of the second peak was recorded and applied in Equation 1 to quantify firmness.

### Ethylene measurements

Ethylene was analyzed on a Focus gas chromatograph (Thermo Electron S.p.A., Milan, Italy) equipped with a FID detector and a RT-QPLOT column, 15 m × 0.53 mm ID (Restek, Bellefonte, PA, USA) at 50°C. Samples of 1 mL were injected using an injection valve. The system was calibrated with a certified gas of 1.01 μL L^−1^ ethylene in synthetic air (Linde Gas Benelux B.V., Schiedam, The Netherlands).

### Peruvian “Kent” mango collection and storage (batch 1)

**Figure 1:**
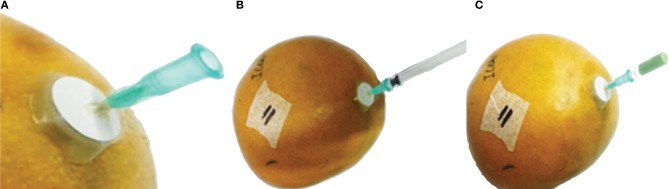
The procedure of extracting ethylene from the seed cavity. **(A)** shows the preparation step where a sticker septum is attached to the distal end of a mango. Stopcock grease, between the septum and the needle, is used to prevent air from leaking out of the seed cavity. **(B)** shows the extraction step where 1 mL of the air from the seed cavity is extracted using a standard 1 mL syringe. **(C)** shows the rubber plug to close the needle until subsequent measurements are scheduled.

Thirty-six “Kent” mangoes of two sizes, size 6 and size 9, with size the number of mangoes per package of 4 kg, were stored individually in 9 L plastic containers (Fibox 12201, Stockton on Tees, UK) at room temperature for 12 days. Containers were opened each day to avoid O_2_ depletion and CO_2_ accumulation. Mangoes within each size class were randomly assigned to two treatments. In the first treatment, mangoes were stored without ethylene. For the second treatment mangoes were stored with the addition of 1 mL pure ethylene in the headspace, equivalent to 115 μL L^−1^ ethylene. The internal ethylene levels were monitored daily by extracting 1 mL gas from the seed cavity. This was accomplished by first attaching a 15 mm diameter septum (Dansensor, Ringsted, Denmark) on the distal end of the mango and then inserting a 1 mL syringe with a 0.8 × 40 mm needle through the septum into the seed cavity. The needle was kept in the mango during the storage period with the open end of the needle closed with a rubber plug to be removed for subsequent measurements (Figure 1). Gas composition, both from the seed cavity and the headspace just before opening the container, was measured daily by gas chromatography.

### Brazilian mango collection and storage (batch 2)

Hundred and twenty Brazilian “Keitt” mangoes were collected in March 2011 from wholesale trader “The Greenery” (Barendrecht, the Netherlands). The fruit were randomly divided into sub-batches of 30 mangoes. Sub-batches were stored at either 10, 17, 24 or 30°C at a constant vapor pressure of 0.21 kPa in Weiss chambers (Weiss Technik, Biochim 1600 S, USA) for 16 days. Repeated firmness measurements on four positions of the fruit, two per mango cheek, took place every 2 days.

### Spanish mango collection and storage (batch 3)

“Keitt” mangoes from an orchard in Velez-Malaga, Spain, were harvested from different positions in the tree [south-high (SH), north-high (NH), south-low (SL) and north-low (NL)]. Three mangoes of similar size and maturity, from one panicle (branched inflorescence), were labeled per position in the tree. The three labeled mangoes on the same panicle were harvested one by one during three consecutive weeks (November 2nd, 9th, and 16th 2012). This setup was applied to fruit from 18 trees, resulting in 72 mangoes per week. After each harvest, the mangoes were put in cardboard boxes and stored at 25°C for 2 days and transported during 3 days at 8°C by truck to Wageningen, The Netherlands. The first half of the mangoes were stored at 20°C for about 2.5 weeks, the other half were stored at 8°C for 3 weeks and subsequently at 20°C for about 10 days. Temperature during transport and storage was recorded using KeyTag KTL-108 dataloggers (Askey Dataloggers, Leiderdorp, The Netherlands). Firmness was repeatedly measured at four positions per fruit, two per mango cheek, starting at harvest, after transport, and then about every 2 to 3 days during storage.

### Puerto Rican mango collection and storage (batch 4)

Forty “Keitt” mangoes were collected from wholesale trader “Bakker Barendrecht” (Ridderkerk, The Netherlands) in July 2012. Fruit were randomly divided into sub-batches of ten mangoes each and stored in temperature-controlled storage rooms at either 12, 16, 20 or 24°C, respectively. Repeated firmness measurements at four positions per fruit, two per mango cheek, were conducted daily for the two highest storage temperatures during 13 days, every 2 days for storage at 16°C during 18 days and every 3 days for storage at 12°C during 22 days.

### Israeli and Brazilian “Keitt” mango collection and storage (batch 5–7)

Two hundred Israeli “Keitt” mangoes were collected from wholesale trader “The Greenery” in October 2012 (batch 5). Fruit were allocated to four sub-batches ranked according to their initial firmness. The firmer sub-batches were stored at higher temperatures whereas the softer sub-batches were stored at lower temperatures with a temperature switch after about 3 days, simulating first ripening and later transport. Mangoes in each sub-batch were allocated randomly to two 40 L plastic low density polyethylene bags, each supplied with a septum. Ethylene was supplied daily by injecting 2.5 mL pure ethylene in the first bag, resulting in 100 μL L^−1^ ethylene in the head space. The mangoes in the second bag were not treated with additional ethylene. Each day every plastic bag was opened for 1 h to avoid O_2_ depletion and CO_2_ accumulation.

Two batches of Brazilian “Keitt” mangoes (80 fruits for batch 6, 74 fruits for batch 7) were collected from “The Greenery” in November 2012. The same ethylene scheme as for the Israeli batches was applied, but now split into two initial firmness classes. Repeated firmness measurements on four positions of the fruit, two per mango cheek, took place about every 1 or 2 days for 10 days (Brazilian batches) or 13 (Israeli batch) days. An overview of the storage conditions of the Israeli and Brazilian batches is provided in Table 1.

**Table 1:** Overview of the average storage conditions and initial firmness for the ethylene treated batches, including the ethylene factor (EF) per sub-batch based on the kinetic parameters of Table 3 and the estimated values of *Eth* (Table 2).

**Cultivar**	**Batch**	**Origin**	**First storage period**		**Second storage period**		**Initial firmness**	**Ethylene**	**EF**
			**Time (days)**	**Temperature (°C)**	**Time (days)**	**Temperature (°C)**	**10^−4^Hz^2^kg^2/3^**	**μL L^−1^**	***k_fenz_ Eth***
Keitt	5a	Israel	2.8	17.8	3.2	13.5	37.4	0	0.013
	5b							100	0.117
	5c		3.2	19.5	10.8	13.0	41.3	0	0.010
	5d							100	0.091
	5e		2.8	16.6	10.2	19.9	44.4	0	0.032
	5f							100	0.286
	5g		3.2	19.8	9.8	22.1	52.3	0	0.056
	5h							100	0.499
Keitt	6a	Brazil	6	20.0	4	20.0	33.8	0	0.062
	6b							100	0.077
	6c		6	16.0	4	20.0	43.6	0	0.035
	6d							100	0.043
Keitt	7a	Brazil	4	18.0	6	16.0	39.0	0	0.056
	7b							100	0.073
	7c		4	16.0	6	20.0	32.0	0	0.083
	7d							100	0.107
Keitt	8a	Spain	10	20	–	–	82.4	0	0.065
	8b						86.2	10	0.113
	8c						86.0	50	0.117
	8d						87.0	100	0.127
	8e						66.9	0	0.065
	8f						69.3	10	0.113
	8g						74.7	50	0.117
	8h						72.8	100	0.127
Keitt	9a	unknown	16	20	–	–		0	0.138
	9b						33.9	3	0.327
	9c							100	0.487
Kent	12a	Ivory Coast		20	–	–		0	0.332
	12b		15				42.8	10	0.408
	12c							100	0.373
Kent	13a	Mali		20	–	–		0	0.285
	13b		15				27.0	10	0.448
	13c							100	0.495

### Spanish “Keitt” mango collection and storage (batch 8)

A batch of 64 Spanish mangoes were collected from wholesaler “Greenyard” (Waddinxveen, The Netherlands) in November 2017. Fruit were ranked according to their initial firmness. Half of the batch, the firmest mangoes, were stored during for 2 days at 13°C, the other half at 20°C to create clear differences in firmness at the start of the experiment. Each half of the batch was divided randomly into four sub-batches, one for each of the ethylene applications (0, 10, 50, and 100 μL L^−1^, Table 1). Ethylene was applied by putting mangoes in plastic bags as described above. Firmness was measured every day for 12 days during storage at 20°C.

### “Keitt” mango collection and storage (batch 9)

Forty-five “Keitt” mangoes of unknown origin were collected from wholesale trader “Bakker Barendrecht” in July 2011. The fruit were randomly divided into three sub-batches and each sub-batch was stored for 16 days in a stainless steel 70 L container at 20°C. The containers were subjected to 0, 0.15 or 5 mL pure ethylene, and resupplied daily, to achieve ethylene levels of 0, 3, and 100 μL L^−1^ in the containers (Table 1). Containers were opened daily for 1 h to avoid O_2_ depletion and CO_2_ accumulation. Just before opening a 1 mL gas sample from each container was taken for GC analysis.

### Brazilian, Malian and Ivory Coast “Kent” mango collection and storage (batch 10–13)

Hundred-twenty Brazilian “Kent” mangoes (batch 10) were collected from wholesale trader “The Greenery” in March 2011. The fruit were randomly divided into four sub-batches and stored in Weiss chambers at either 10, 17, 24 or 30°C at a constant vapor pressure of 0.21 kPa for 16 days. Eighty Malian “Kent” fruits (batch 11) were collected from wholesaler “The Greenery” in July 2012 and stored at either 12, 16, 20 or 24°C in storage rooms.

Sixty Ivory Coast (batch 12) and 80 Malian “Kent” mangoes Malian fruit (batch 13) were collected from wholesaler “The Greenery” in September 2016. The firmest 30 mangoes per batch were randomly divided into three sub-batches and stored in stainless steel 70 L containers at 20°C. The containers were treated with either 0, 10 or 100 μL L^−1^ ethylene (Table 1). Containers were opened daily for 1 h and resupplied with ethylene after closing. Repeated firmness measurements on four positions of the fruit, two per mango cheek, took place every 1 or 2 days.

## Model development

### Model formulation

A kinetic model is developed that describes the mango softening behavior. The model is linked as closely as possible to the softening physiology, but also sufficiently simple to allow analysis of datasets varying in sourcing region, cultivar, storage temperature, and exogenous ethylene application. Firmness (*F*) is assumed to be related to structural cell wall compounds that are broken down by a complex of enzymes (*Enz*) as described in the introduction. Cell wall breakdown can then be described according to Equation 2 with *k*_*f*_ (in d^−1^) the rate constant for the softening.

(2) $$\begin{array}{l} F+Enz\overset{k_{f}}{\underset{\;}{\to }}Enz \end{array}$$

The enzyme system (*Enz*) is assumed to be induced by ethylene (*Eth*) approximated by Equation 3 with *k*_*enz*_ (in d^−1^) the rate constant for enzyme production. Equation 3 assumes the ethylene level remains constant over time.

(3) $$\begin{array}{l} Eth\overset{k_{enz}}{\underset{\;}{\to }}Eth+Enz \end{array}$$

As for most enzyme systems, it is assumed that there is a turnover of the cell wall degrading enzymes between enzyme production (Equation 3) and the enzyme degradation (Equation 4) with *k*_*d*_ (in d^−1^) the rate constant of enzyme degradation.

(4) $$\begin{array}{l} Enz\overset{k_{d}}{\underset{\;}{\to }}nil \end{array}$$

The model, described as a number of coupled processes, can mathematically be presented as a set of ordinary differential equations [Equations (5–7)], based on the rules of chemical kinetics. *F*_*fix*_ is the residual, basic firmness level representing the final asymptotic firmness level.

(5) $$\begin{array}{l} \frac{\partial }{\partial t}\left[Eth\right]=0 \end{array}$$

(6) $$\begin{array}{l} \frac{\partial }{\partial t}\left[Enz\right]=k_{enz}\left[Eth\right]-k_{d}\left[Enz\right]\text{with}\left[Enz\right]\left(t=0\right)={Enz}_{0} \end{array}$$

(7) $$\begin{array}{l} \frac{\partial }{\partial t}\left[F\right]=-k_{f}\left[F-F_{fix}\right]\left[Enz\right]\text{with}\left[F\right]\left(t=0\right)=F_{0} \end{array}$$

An analytical solution exists for this mechanism [Equations (5–7)]: (Equation 8).

(8) $$\begin{array}{l} F=F_{fix}\\ +\left(F_{0}-F_{fix}\right)\;e^{k_{f}\left(\frac{\left(Enz_{0}k_{d}-k_{enz}Eth\right)e^{-k_{d}t}}{{k_{d}}^{2}}-\frac{k_{enz}k_{d}Etht+k_{enz}Eth-k_{d}Enz_{0}}{{k_{d}}^{2}}\right)} \end{array}$$

All three reaction rate constants (*k*_*enz*_, *k*_*f*_ and *k*_*d*_) depend on temperature (*T*, in K) assumed according to Arrhenius' Law [Equation (9)].

(9) $$\begin{array}{l} k_{i}=k_{i,ref}e^{\frac{E_{i}}{R}\left(\frac{1}{T_{ref}}-\frac{1}{T}\right)} \end{array}$$

The reaction rate constant *k*_*i, ref*_ is the value of *k*_*i*_ at an arbitrary chosen reference temperature *T*_*ref*_ (here 295.15 K or 22°C), and *E*_*i*_ the energy of activation (in kJ mol^−1^) for the ith reaction. *R* is the universal gas constant (8.314 J mol^−1^ K^−1^).

### Statistics

The equations of the model formulation [Equations (2–4)] were developed using Maple 2017 (MapleSoft, Waterloo Maple Inc., Waterloo, Canada). The developed model was implemented in two ways, both assuming that the *FI* values obtained from the AFS (Equation 1) directly relate to the firmness *F* used in the model formulation. With the first method the ordinary differential equations [ODEs, Equations (5–7)] are directly used in OptiPa (Hertog et al., 2007a), a dedicated tool for data analysis using differential equations. A variable-step continuous solver, ODE45 with a termination tolerance of 0.001 was applied. This method was used for variable temperature scenarios, such as the combined “Keitt” dataset (batches 2–3) and the second Spanish “Keitt” dataset (batch 8). The second method was applied to save time as the ODE implantation takes several days to run per batch. This method was applied for all datasets gathered at constant external conditions using the analytical solution [Equations (8, 9)]. This method analyzed firmness data over time at (constant) temperatures using an indexed version of the nls (non-linear regression) procedure of R (R Core Team, 2016). Both methods allow assigning variation in the experimental data to specific sources. The variation in firmness data was estimated per individual mango (*F*_0_) or estimated in common. Common parameters were estimated either per cultivar (the rate constants) or per batch (*F*_*fix*_ and *Eth*).

When indicated, results were compared applying analysis of variance (ANOVA) using Genstat 18th edition (VSN international Ltd., Hemel Hempstead, UK). Means were compared by the least significant difference (Fisher's protected LSD) test at *P* < 0.05.

## Results

Two types of experiments were setup. The first type investigated the key assumption of the model setup, namely that the internal ethylene level during storage is constant over time. Also, the effect of adding ethylene in the headspace was investigated to examine mango ethylene permeability. The second type of experiments examined the firmness loss for a number of batches with and without application of ethylene and as a function of temperature to estimate both kinetic and initial parameters of the softening model. In total, 12 mango batches were collected over 6 years with an overview provided in Table 2. All batches were collected from wholesale traders, except for batch 3 which was harvested and collected in Spain. All batches were transported by regular reefer transport except for the Spanish batches that were transported by truck. Batches were either divided into sub-batches, each treated at a constant temperature, varying between 10 and 30°C, or stored at variable temperature treatments to simulate postharvest chain scenarios. Most batches were stored at 20 °C and divided into sub-batches, each treated with daily dose of ethylene, varying between 0 and 100 μL L^−1^.

**Table 2:** Overview of the mango batches.

**Cultivar**	**Batch**	**Origin**	**Temperature**	**Ethylene**	***F_0_* (10^-4^ Hz^2^ kg^2/3^)**		***Eth***		***F_fix_* (10^-4^ Hz^2^ kg^2/3^)**		**$R_{\text{adj}}^{2}$**
			**°C**	**μL L^−1^**	**Average**	**St.dev**.	**Estimate**	**S.E**.	**Estimate**	**S.E**.	**%**
Kent	1	Peru	20	0	33.0	3.9	–	–	–	–	–
				115							
Keitt	2	Brazil	10,17,24,30	0	61.7	16.8	1.52	0.03	12.7	0.3	94.9
Keitt	3	Spain	variable	0	43.8	4.6	0.83	0.05	3.7	0.7
Keitt	4	Puerto Rico	12,16,20,24	0	50.4	8.7	0.80	0.03	23.7	0.3	86.0
Keitt	5	Israel	variable	0	43.1	6.2	0.63	0.02	18.2	0.3	89.1
				100			5.62	0.27			
Keitt	6	Brazil	variable	0	39.1	6.5	1.01	0.06	13.5	0.8	90.0
				100			1.25	0.09			
Keitt	7	Brazil	variable	0	35.9	4.4	1.97	0.12	16.4	0.4	89.4
				100			2.54	0.15			
Keitt	8	Spain	variable	0	82.4	1.3	1.05	0.03	17.8	0.4	94.8
				0	66.9	1.4					
				10	86.2	1.4	1.83	0.06			
				10	69.3	1.6					
				50	86.0	1.4	1.89	0.07			
				50	74.7	1.7					
				100	87.0	1.5	2.06	0.07			
				100	72.8	1.7					
Keitt	9	unknown	20	0	33.9	11.1	2.23	0.36	7.6	0.4	87.4
				3			5.30	0.81			
				100			7.89	0.84			
Kent	10	Brazil	10,17,24,30	0	36.3	12.5	0.48*	0.13	6.9	0.5	95.0
Kent	11	Mali	12,16,20,24	0	34.2	14.4	0.76*	0.18	22.6	0.5	
Kent	12	Ivory Coast	20	0	42.8	2.6	0.57*	0.14	8.0	0.8	
				10			0.70*	0.16			
				100			0.64*	0.15			
Kent	13	Mali	20	0	27.0	3.4	0.49*	0.14	7.6	0.7	
				10			0.77*	0.22			
				100			0.85*	0.23			

*Batch 1 is used for measuring internal ethylene levels. The estimated values for F_0_, Eth, F_fix_, and the adjusted variance accounted for (${\text{R}}_{\text{adj}}^{\text{2}}$) are presented for batch 2-13. ${\text{R}}_{\text{adj}}^{2}$ is reported for estimating both kinetic parameters (Table 3) and initial conditions for batches 2 and 3 for “Keitt” and batches 10-13 for “Kent” mangoes. For all other batches, ${\text{R}}_{\text{adj}}^{2}$ is reported with fixed kinetic parameters, estimating F_0_ per mango and Eth per batch*.

-, not estimated * estimates applying the partial set of “Kent” kinetic parameters.

### Internal ethylene levels of the Peruvian mango batch

**Figure 2:**
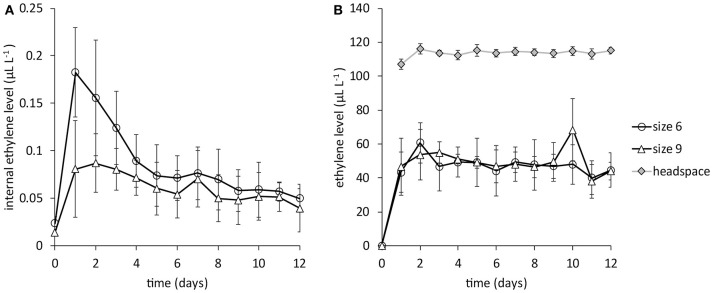
Internal ethylene levels for big (size 6) and small (size 9) mangoes from batch 1, the Peruvian “Kent” batch (*N* = 6). **(A)** the internal ethylene level without ethylene application. **(B)** the internal ethylene level when 1 mL pure ethylene is added in the headspace. Note the difference in ethylene levels in Figures 1A,B.

Internal ethylene levels, without ethylene application, initially increased after the first gas extraction, then leveled off to very low levels around 0.05 μL L^−1^ for both large (size 6, around 670 g) and small mangoes (size 9, around 450 g) until the last days of measurements (Figure 2A). With daily application of 1 mL pure ethylene into the headspace the internal ethylene level increased to around 50 μL L^−1^ in 24 h. The ethylene level in the headspace was around 115 μL L^−1^, 2.4 times higher than observed in the stone, regardless of mango size (Figure 2B).

### Estimating kinetic parameters for the “Keitt” firmness model

Data from the prior reefer transported Brazilian batch (batch 2) and a freshly harvested Spanish batch (batch 3) were combined with the aim to estimate a set of kinetic parameters for “Keitt” mangoes independent of sourcing region. Due to the dynamic temperature scenarios present in the experimental setup of the Spanish batch the ODE formulation of the model [Equations (5–7)] was applied. During the first step of the analysis, all model parameters were estimated in common. In subsequent steps variation was attributed on batch level (*F*_*fix*_, *Eth* and *Enz*_0_) and on individual fruit level (*F*_0_). That means that one value for *F*_*fix*_, *Eth* and *Enz*_0_ was estimated for each batch, and one value for *F*_0_ for each individual fruit. The estimated values for *Enz*_0_ were very small for both batches with large standard deviations. Therefore, *Enz*_0_ was fixed to zero. This simplification has consequences for the model formulation of Equation (8) as now *k*_*f*_ and *k*_*enz*_ are always present together, preventing separate estimation. Therefore, *k*_*f*_ and *k*_*enz*_ were replaced by *k*_*fenz*_, the combined reaction constant, *k*_*f*_ times *k*_*enz*_, that describes the interaction of the enzyme system with the cell wall compounds. This results in the final formulation of the firmness model [Equation (10)] applied during data analysis for both cultivars, together with Equation (9) to account for variation in storage temperature. *k*_*fenz*_ and *Eth* are also always present together in Equation (10). *k*_*fenz*_ times *Eth* indicates how fast the enzyme system responds to ethylene, or in other words, the ethylene factor (EF).

(10) $$\begin{array}{l} F=F_{fix}+\left(F_{0}-F_{fix}\right)\;e^{k_{fenz}Eth\left(\frac{-e^{-k_{d}t}}{{k_{d}}^{2}}-\frac{k_{d}t+1}{{k_{d}}^{2}}\right)} \end{array}$$

**Figure 3:**
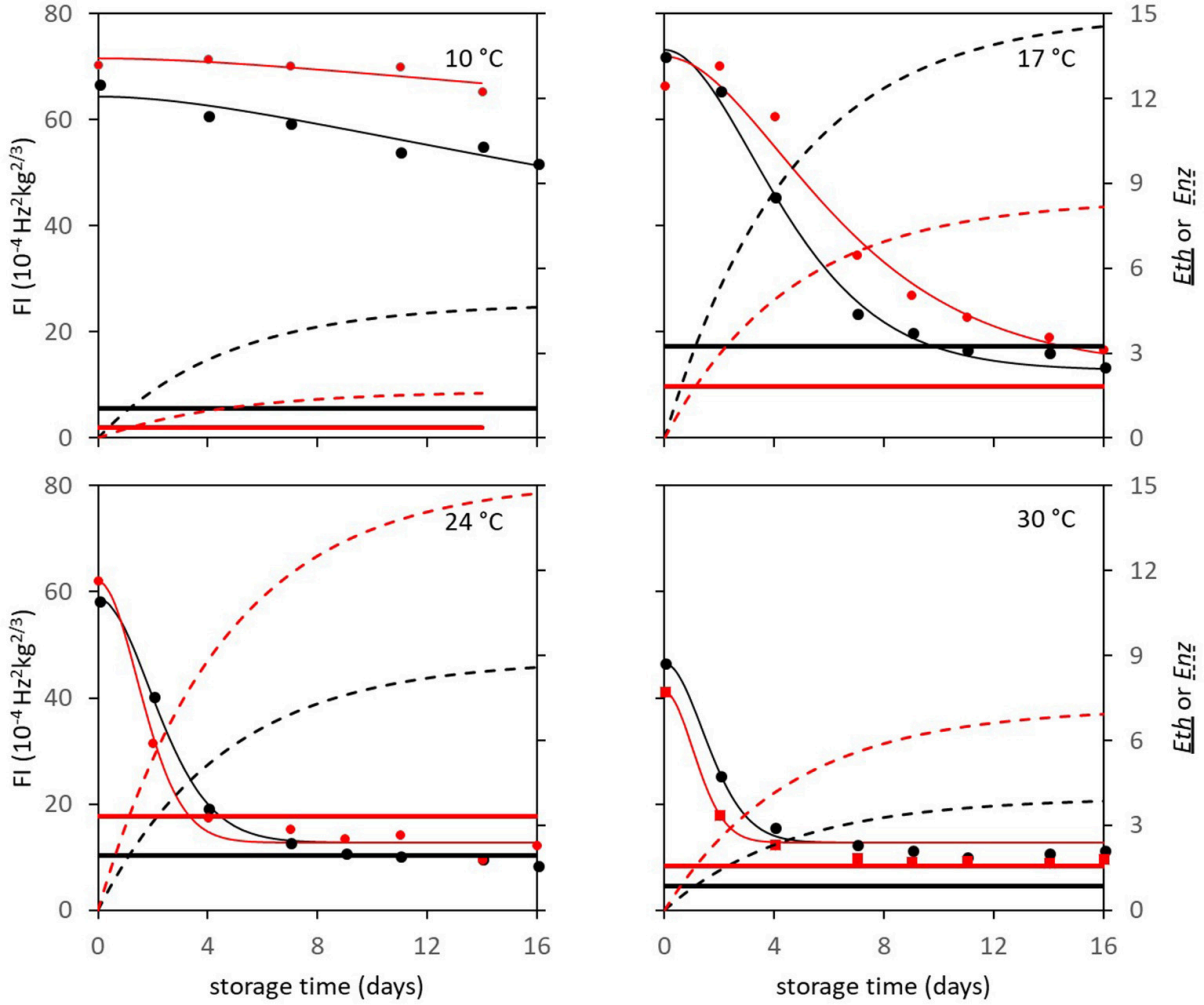
Experimental (points) and simulated (bold lines) firmness behavior of Brazilian “Keitt” mangoes of batch 2, two (one in black and one in red) mangoes for each of the indicated storage temperatures. The simulated lines are based on the kinetic parameters in Table 3. Also, both *Eth* (double lines) and *Enz* (dotted lines) per mango are simulated based on the parameters in Table 1.

**Figure 4:**
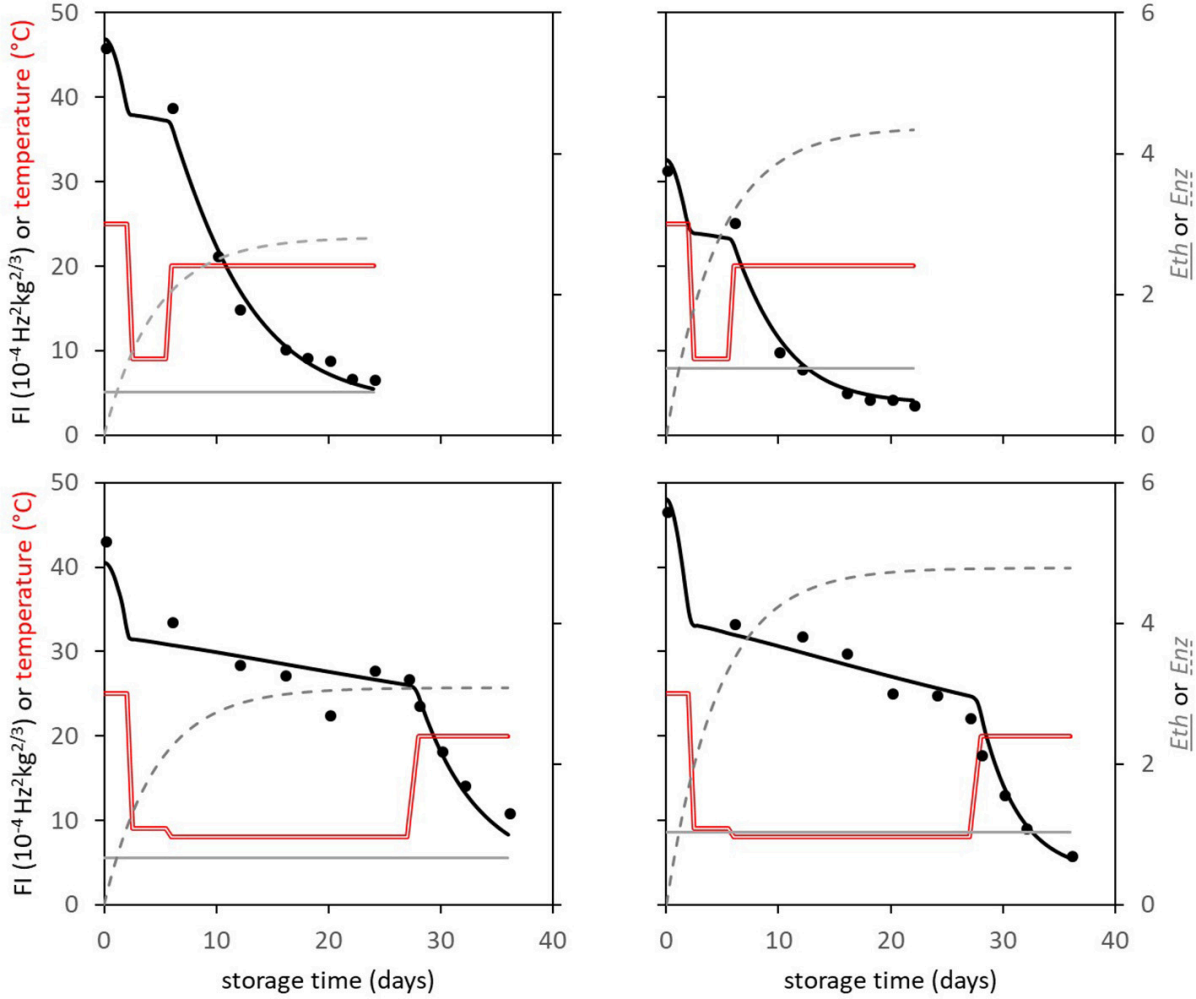
Experimental (points) and simulated (bold black lines) firmness behavior of four Spanish “Keitt” mangoes of batch 3. Temperature is indicated by the double red lines. The simulated lines are based on the kinetic parameters in Table 3. Also, both *Eth* (gray solid lines) and *Enz* (gray dotted lines) per mango are simulated based on the parameters shown in Table 1.

In subsequent analysis steps the kinetic parameters *F*_*fix*_ and *Eth* were estimated per batch, while *F*_0_ was estimated per individual mango. The standard error of estimates were estimated smaller than 10% of the parameter estimates (Table 3), with a high percentage variance accounted for (Table 2). In the final step *Eth* was also estimated per individual mango. Examples of the firmness data and simulation for two Brazilian mangoes (batch 2), similar in initial firmness, are shown per storage temperature in Figure 3. Figure 4 shows measured and simulated firmness behavior for one Spanish mango (batch 3) per temperature scenario.

**Table 3:** Estimated model parameters for both cultivars.

**Cultivar**	**Kinetic parameters**			
		**Units**	**Estimate**	**St.dev**.
	*k_*fenz, ref*_*	mol^−1^ d^−1^	0.099	0.007
	*k_*d, ref*_*	d^−1^	0.219	0.034
Keitt	*E_*fenz*_*	kJ mol^−1^	169.9	3.6
	*E_*d*_*	kJ mol^−1^	0.01	8.2
	*k_*fenz, ref*_*	mol^−1^ d^−1^	1	set
	*k_*d, ref*_*	d^−1^	1.98	0.56
Kent	*E_*fenz*_*	kJ mol^−1^	194.5	17.6
	*E_*d*_*	kJ mol^−1^	162.2	20.3
	*T_*ref*_*	°C	22	

**Figure 5:**
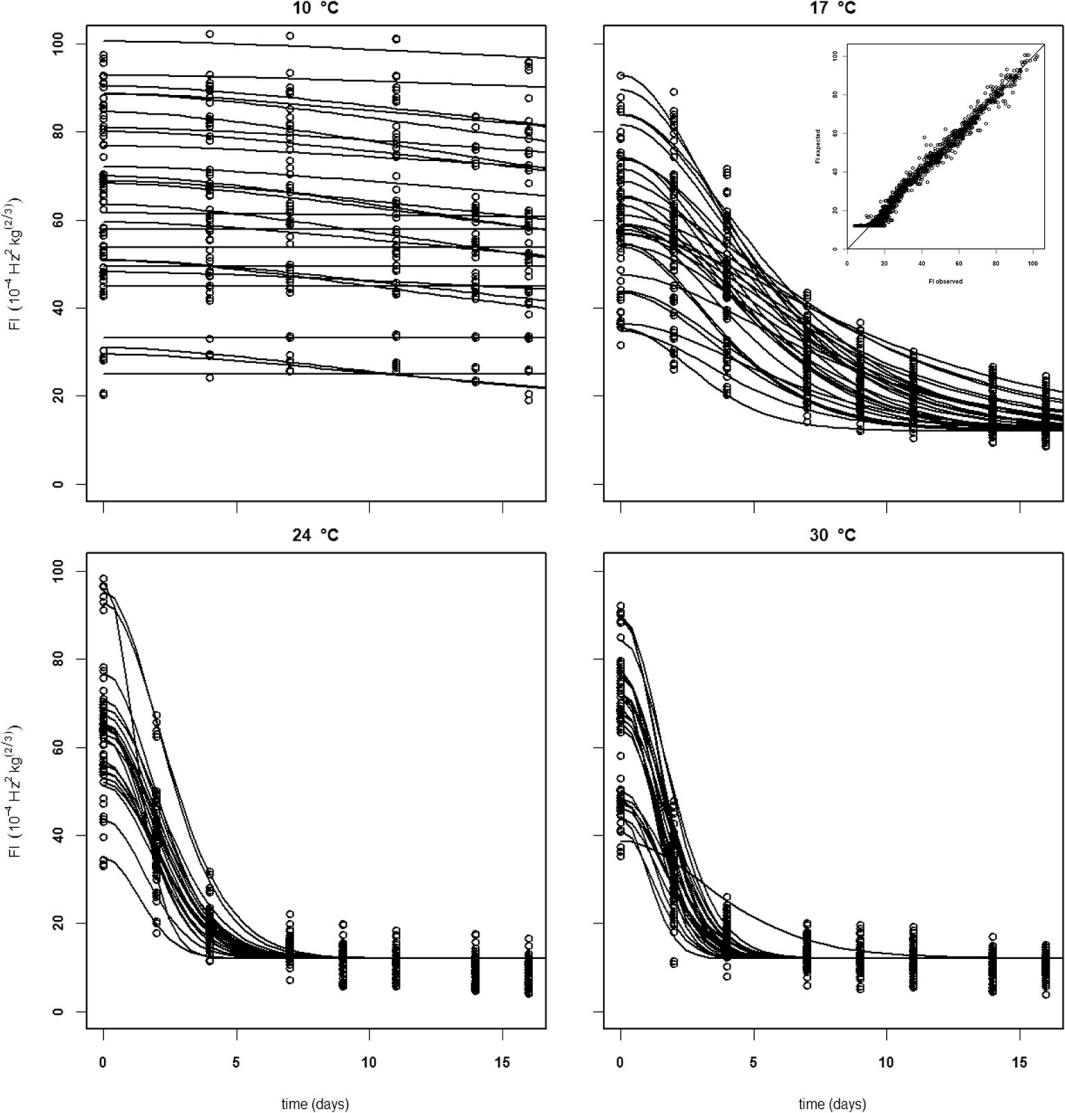
Experimental (black dots) and simulated (lines) firmness behavior of thirty Brazilian “Keitt” mangoes (batch 2) per indicated temperature, applying fixed kinetic parameters from Table 3 and only estimating *Eth* and *F*_0_ per mango. Inset shows the simulated vs. measured firmness values for the whole batch.

**Figure 6:**
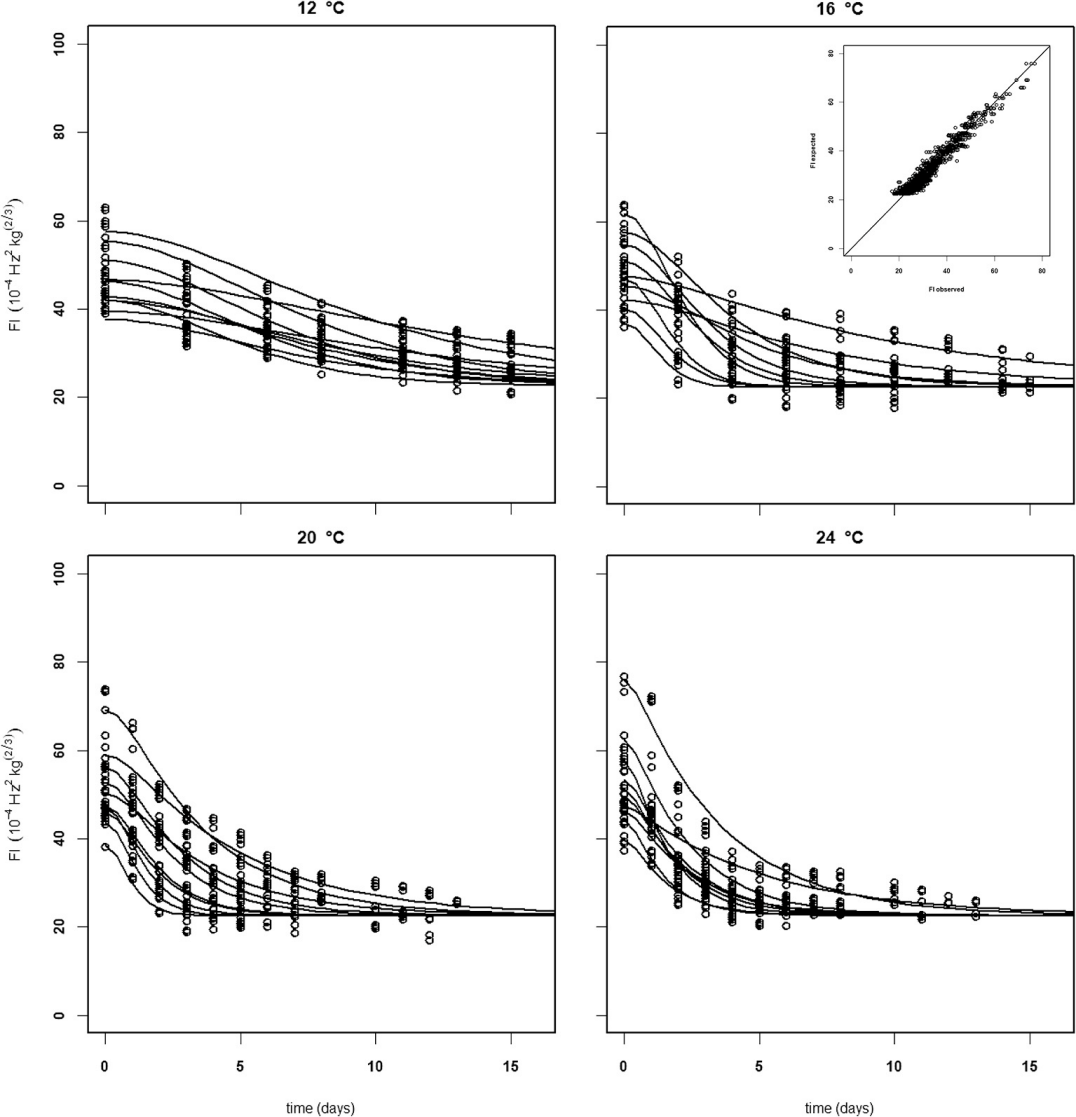
Experimental (black dots) and simulated (lines) firmness behavior of 10 Puerto Rican “Keitt” mangoes (batch 4) per indicated temperature, applying fixed kinetic parameters from Table 3 and only estimating *Eth* and *F*_0_ per mango. Inset shows the simulated vs. measured firmness values for the whole batch.

For the analysis of the Puerto Rican “Keitt” batch (batch 4, Table 2) the kinetic parameter set estimated on the Spanish and Brazilian mangoes was used, estimating only the initial parameters (*Eth* and *F*_*fix*_ per batch and *F*_0_ per mango). This shows that this kinetic parameter set can be transferred to another batch. Remarkably, the estimated value for the final firmness (*F*_*fix*_) of “Keitt” batches 2 and 4 shows a difference of about 11 FI (Figures 5, 6).

### Estimating kinetic parameters for “Kent” mangoes

**Figure 7:**
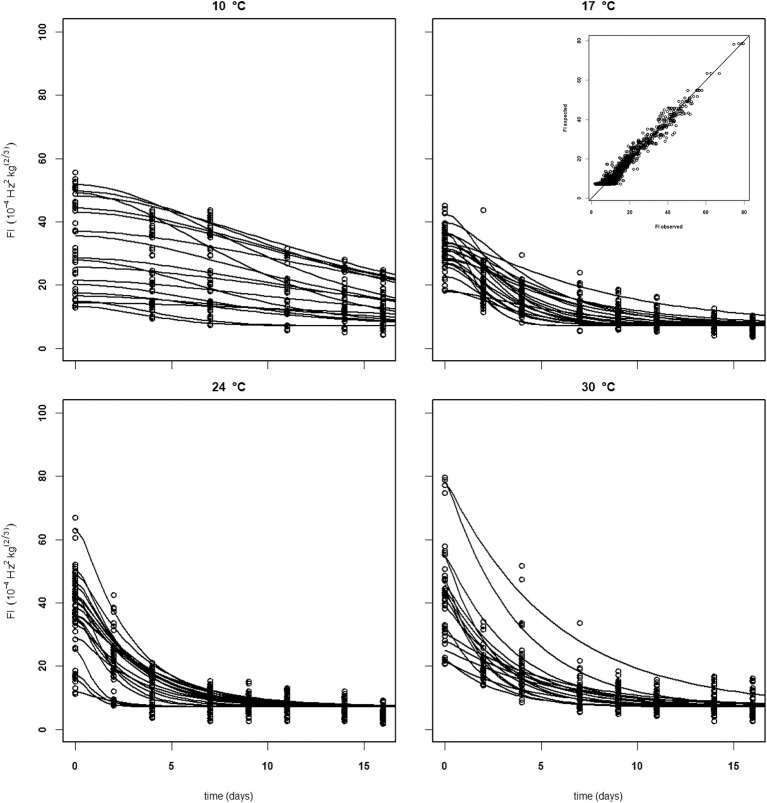
Experimental (black dots) and simulated (lines) firmness behavior of thirty Brazilian “Kent” mangoes (batch 10) per indicated temperature, applying fixed kinetic parameters from Table 3 and estimating only *Eth* and *F*_0_ per mango. Inset shows the simulated vs. measured firmness values for the whole batch.

Establishing a set of kinetic parameters for “Kent” mangoes was more difficult because “Kent” mangoes are, at the start of the experiment, considerably less firm than “Keitt” mangoes (Table 2). For “Kent” mangoes all firmness data from four batches (batches 10–13), stored without ethylene were combined. This combined dataset was analyzed applying the final firmness model [Equations (9, 10)]. Nevertheless, an acceptable set of kinetic parameters could not be estimated. To be able to estimate a (partial) kinetic parameter set for “Kent” mangoes the approach was taken to estimate the combined value for *k*_*fenz*_ and *Eth*, EF. An acceptable set of kinetic parameters could be estimated, although with standard deviations a factor two to three times higher than for “Keitt” mangoes (Table 2). Measured and simulated firmness for all “Kent” mangoes of batch 10, applying the kinetic parameters of Table 3 and estimating *F*_0_ and *Eth* per mango, is shown in Figure 7. The variation in *F*_*fix*_ found in “Keitt” batches is also visible for “Kent” bathes, for instance when comparing batch 10 and batch 11 which differ about 16 FI (Table 1).

### The ethylene factor is affected by position in the tree

**Figure 8:**
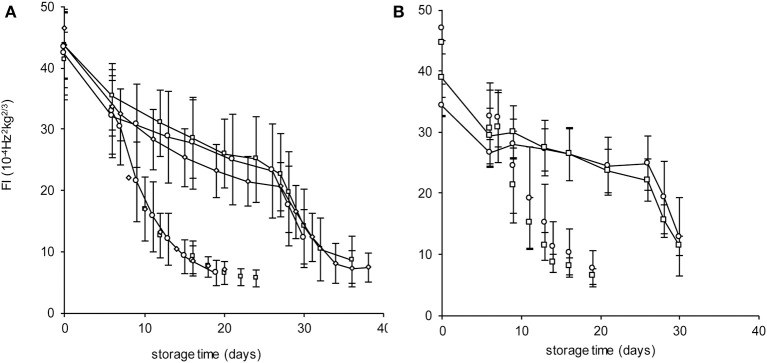
Firmness behavior with indicated standard deviation over time for the Spanish “Keitt” mangoes of batch 3 for the high temperature scenario (dashed lines) or the low temperature scenario (solid lines) as shown in Figure 4. **(A)** the firmness behavior over time of mangoes harvested during the three-week harvest window (week 1: squares, week 2: diamonds, week 3: circles). **(B)** the firmness behavior over time for mangoes harvested from the NH (circles) and SL (squares) tree positions in week 3.

**Figure 9:**
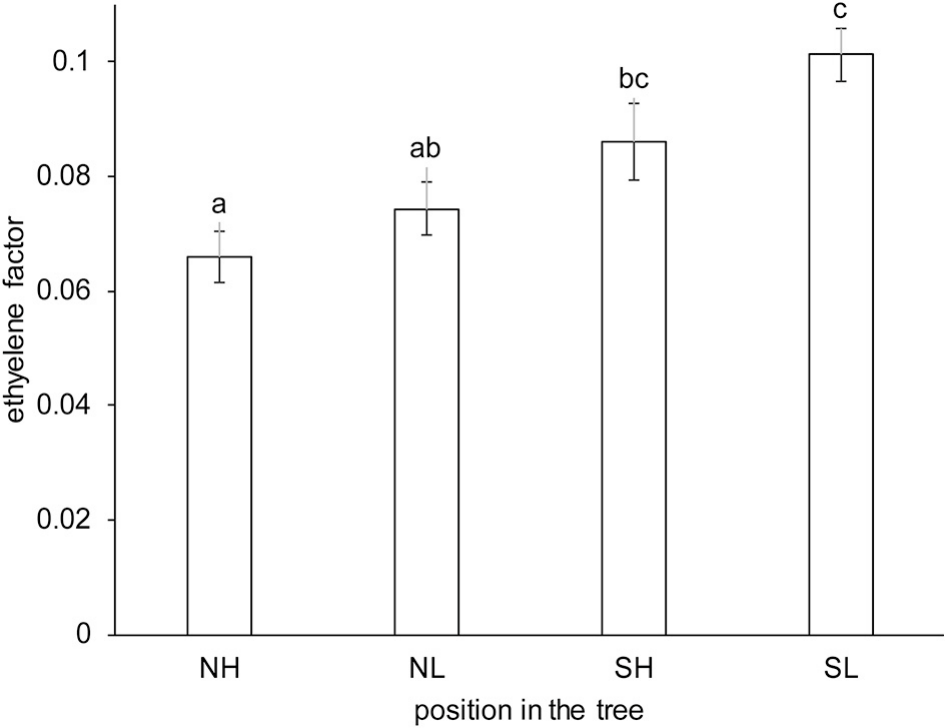
Estimated average ethylene effect (*k*_*fenz*_ times *Eth*) with standard deviation indicated for the Spanish “Keitt” mangoes (batch 3) classified per location in the tree. NH indicates mangoes harvested from the northern and upper part, NL from the north and lower part, SH from the southern and upper part and SL from the southern and lower part of the tree. The kinetic parameters from Table 3 were applied, only estimating *Eth* per position in the tree and the initial firmness per mango, with a total of 216 mangoes from three trees. The same letter indicates no significant difference, while different letters indicate significant differences (*P* < 0.05).

The Spanish “Keitt” mangoes (batch 3) were harvested three times in a 3 weeks harvest window and firmness was measured directly after harvest and during a low and high temperature scenario (Figure 8A). The effect of harvesting from different parts of the tree canopy [south-high (SH), north-high (NH), south-low (SL) and north-low (NL)] was investigated by estimating the initial firmness (*F*_0_) and EF (*k*_*fenz*_ times *Eth*) as function of the position in the tree, applying the final model formulation [Equations (9, 10)]. No effect was found of canopy location on the initial firmness. A small, but significant, effect was found for EF, with mangoes from the SL quarter showing the highest, and from the NH quarter showing the lowest level (Figure 9). This indicates slightly faster softening for SL-, and slightly slower softening for NH harvested mangoes as shown for mangoes harvested in week 3 (Figure 8B).

### Ethylene application shows high variability with regard to mango softening

**Figure 10:**
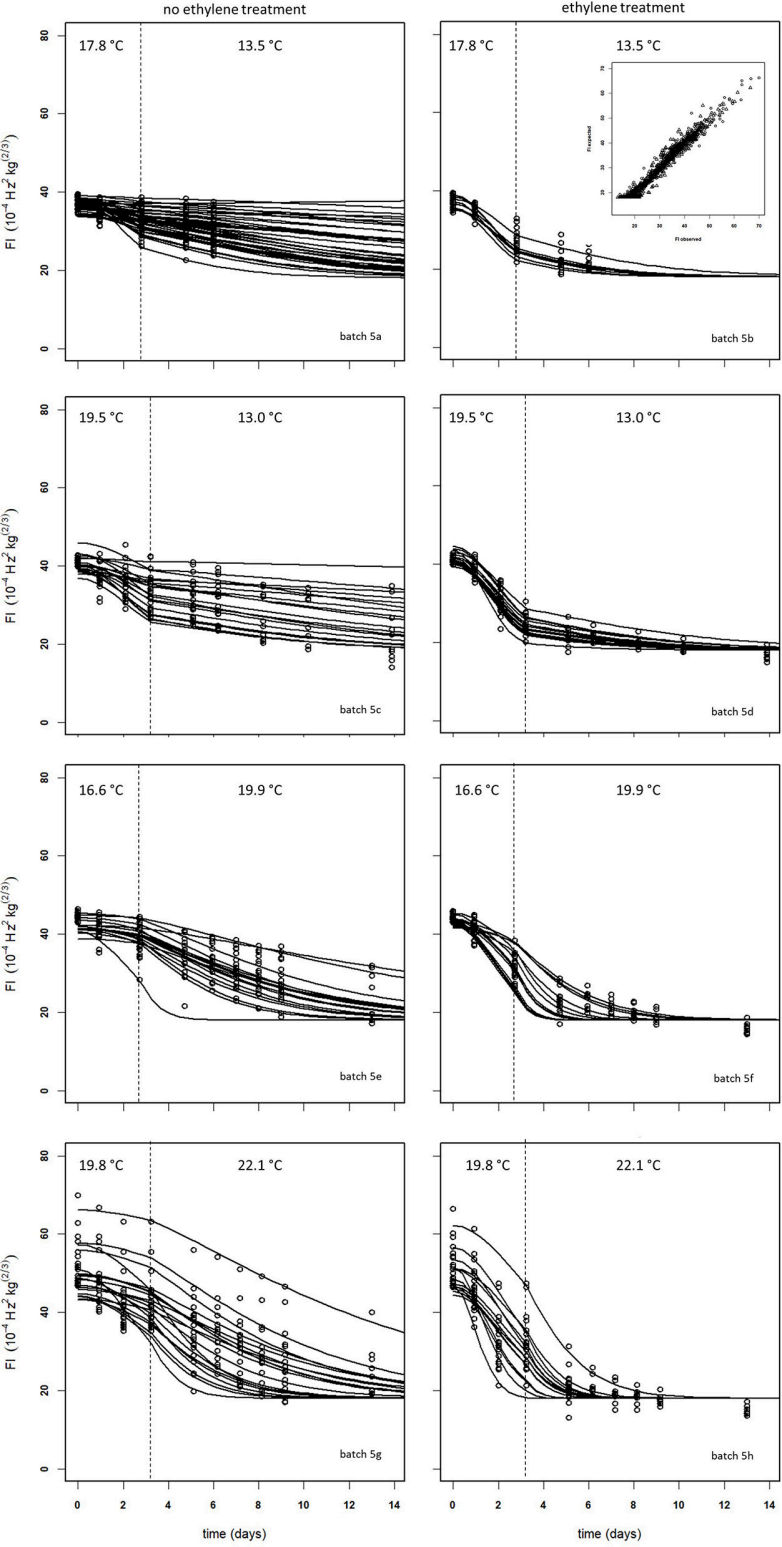
Experimental (black dots) and simulated (lines) firmness behavior for the Israeli “Keitt” sub-batches 5a–h (Table 1) treated without (left column) and with (right column) ethylene, applying fixed kinetic parameters from Table 3 and estimating *F*_*fix*_ per batch and *Eth* and *F*_0_ per mango. The dotted lines in each plot indicate the temperature switch after about 3 days. Inset shows the simulated vs. measured firmness values for the whole batch.

Figure 10 shows experimental and simulated data for the Israeli “Keitt” batch (batch 5) with (right column) and without (left column) 100 μL L^−1^ external ethylene application. Ethylene application has a clear effect as the measured firmness behavior always decreases sharply per temperature scenario, for instance when the softening of mangoes in sub-batches 5e and 5f are compared. The ethylene induced softening is linked with the storage temperature, as higher temperature scenarios show increased softening, for instance when comparing the softening behavior of sub-batches 5c and 5e. Higher ethylene induced softening results in substantial reduction in the observed firmness variation (Figure 10).

The effect of external ethylene application per batch was quantified by estimating *Eth* per ethylene treatment and the initial firmness per individual mango (Table 2). Kinetic parameters were fixed and applied as reported in Table 3 for either “Keitt” or “Kent” mangoes. The ethylene factor (EF, *k*_*fenz*_ times *Eth*) was calculated, with *Eth* taken from Table 2 and *k*_*fenz, ref*_ and *E*_*fenz*_ taken from Table 3 to calculate *k*_*fenz*_ according to average temperature during the storage period(s) (Table 1). External ethylene application of 100 μL L^−1^ for the Israeli “Keitt” batch (batch 5) resulted in an EF almost a factor nine higher than without ethylene application for each temperature scenario (Table 1). When EF is compared between temperature scenarios, then EF for the ethylene treated sub-batch at the highest storage temperature (sub-batch 5h) is almost fifty times higher than that for the non-ethylene treated sub-batch at the lowest average storage temperature (sub-batch 5c) (Figure 10). This indicates that also temperature has a major impact on EF. This temperature effect is larger for “Kent” than for “Keitt” mangoes since the activation energy *E*_*fenz*_ is higher for “Kent” mangoes (Table 3).

Applying 100 μL L^−1^ of external ethylene to the “Keitt” mangoes of batch 8 resulted in almost doubling EF compared to the non-ethylene treatment. Applying a low ethylene level (10 μL L^−1^) already provided for the major part of the response in terms of increasing EF. “Keitt” mangoes from batch 9 also showed a substantial EF as response to external ethylene. Even with a low level of externally ethylene applied (3 μL L^−1^) EF more than doubled, reaching 350% when 100 μL L^−1^ was applied than without ethylene application (Table 1). Ethylene application resulted in a clear response by at least doubling EF for “Keitt” batches 5, 8, and 9. However, this was quite different for the Brazilian “Keitt” batches (batches 6, 7). Here, also faster ripening was observed by having higher EFs, but now for only 20-30% (Table 1) compared to the non-ethylene treatments. For “Kent” batches EF seems to be non-existent (batch 12) to small (batch 13), increasing EF with only 60% compared to control (Table 1).

## Discussion

### Internal ethylene levels are likely constant

Without applying external ethylene, internal ethylene levels are very low for Peruvian mangoes (Figure 2A) after 24 h and appear to be constant over time. This supports the assumption used in the model development [Equation (5)] that for prior transported mangoes the ethylene level is constant. After the climacteric peak, ethylene keeps being produced, although at a very low level (Zaharah et al., 2013). Likely, ethylene is required for further ripening in climacteric fruit as 1-MCP treatment applied during the ripening rapidly decreased the mRNA levels of phytoene synthase 1, expansin 1, and ACC oxidase, even in red ripe tomato fruit (Hoeberichts et al., 2002). This low level of ethylene production might be counterbalanced by ethylene loss through the skin leading to a low and constant level of ethylene in the fruit that determines the actual production and activity of softening enzymes.

When internal ethylene levels are very low, application of ethylene might have a drastic effect on internal ethylene levels. The ethylene level in the seed cavity increased quickly after ethylene application, but not to the level as measured in the headspace. The seed cavity ethylene level after 24 h was fairly constant over time, a factor 2.4 times lower than in the headspace (Figure 2B). However, the daily opening of the boxes likely prevented the internal ethylene level to catch up with that in the headspace. It is therefore unlikely that the lack of increased softening due to external ethylene observed in a number of batches (Table 1) is due to limited ethylene permeability.

### The physiological shortcuts of the firmness model

Zaharah et al. (2013) reported that mangoes show a sharp peak in ethylene production about 2 to 3 days after harvest. This induced the production of endo-polygalacturonase reaching a maximum 1 to 2 days later followed by a decrease in production. This might indicate that fully mature mangoes on the tree exhibit maximal firmness due to lack of production of cell wall degrading enzymes. It is likely that the initial level of the enzyme system, i.e., the value of *Enz*_0_, is very small. The assumption to set *Enz*_0_ to zero, as used during the estimation of the kinetic parameters (section Estimating Kinetic Parameters for the “Keitt” Firmness Model), might therefore be valid.

There is a large difference between the measured internal ethylene level without and with external ethylene application for the Peruvian batch (batch 1), about a factor 500 (section The Physiological Shortcuts of the Firmness Model). The ratio between the maximum estimated value of *Eth* with and without ethylene application found for the Israeli batch (batch 5) is only a factor nine. It is therefore clear that the *Eth* values do not reflect the internal ethylene levels and need to be interpreted in terms of EF (*Eth* times *k*_*fenz*_, Table 1). The physiological interpretation is then that EF for the Israeli batch is nine times higher when ethylene is applied compared to no ethylene application. From a physiological viewpoint EF is a useful concept as it is a reflection of both internal ethylene level and ethylene sensitivity. The nature of *Eth* needs to be clarified in future research. The link between EF and the measured internal ethylene levels needs to be clarified. Also, EF is affected by the position in the tree (Figure 9) that would indicate that growth conditions play a role (see below), but EF might also be affected by handling, such as hot water treatments and early ethylene exposure during handling in the chain.

### Chilling might be limiting softening enzyme activity

Reefer transported mangoes are refrigerated between 8 and 14°C (Hamburgsüd., 2016) for several weeks. This might initiate chilling injury, such as inhibition of polygalacturonase and β-galactosidase activity (Ketsa et al., 1999). Cold storage also affects the activity of numerous cell wall degrading enzymes in peach (Brummell et al., 2004), and especially that of endo-polygalacturonase (Lurie and Crisosto, 2005), also important in fruit softening. This effect of chilling on softening is not modeled explicitly [Equations (5–7)]. It is however, interesting that the value for *E*_*d*_, the energy of activation for the enzyme degradation, varies greatly between cultivars. The large and positive value found for “Kent” mangoes indicates faster enzyme breakdown at higher temperatures. For “Keitt” mangoes the value of *E*_*d*_ is very close to zero (Table 3). This might indicate that “Keitt” mangoes are sensitive to enzyme decay at lower temperatures, a chilling injury symptom. This would result in much reduced softening enzyme levels for “Keitt” compared to “Kent” mangoes during cold reefer transport. This is reflected in higher initial firmness for “Keitt” batches at the start of the experiments (Table 1). The suggestion that “Keitt” mangoes suffer from chilling affecting softening seems to be confirmed by Nair and Singh (2009) that linked chilling injury to firmness retention in “Kensington Pride” mangoes.

### The tree factor might be affected by growth conditions

In a number of fruit crops (e.g., mango, avocado and some apple cultivars) fruit ripening is delayed as long as the fruit is attached to the tree. This phenomenon is called the tree factor (Burg and Burg, 1965; Blanpied, 1993; Lin and Walsh, 2008). Avocado shows the strongest tree factor effect as it inhibits the ripening process for almost a year. During the entire time when the fruit is attached to the tree no climacteric ethylene is produced (Lin and Walsh, 2008). It seems, therefore, that harvesting is a pivotal event for the softening behavior. Indeed, mangoes harvested from the same panicle during the 3 weeks harvest window showed on average the same initial firmness (Figure 8A), indicating that softening in the tree was very slow compared to that after harvest. The nature of the tree factor is currently unknown. It might be hypothesized that the tree factor is gaseous compound that diffuses out of the mango once harvested, but that when attached inhibits ethylene production. A small, but significant, effect was found for EF as affected by position in the tree (Figure 9). As EF varied with position in the tree, and the tree factor inhibits ethylene production, it might indicate that the tree factor is affected by light and temperature during the growing period. Elucidating the nature of the tree factor might be beneficial for the mango chain as it might allow transport of mangoes that normally would otherwise soften substantially. This would include “Kent” mangoes that show significant lower firmness than “Keitt” mangoes at the arrival in the Netherlands. Application of the tree factor compound might also allow reefer transport of mangoes from cultivars that are cultivated in e.g., India and Pakistan that currently reach Europe only by air transport.

### Variation in the ethylene factor might be due to chilling

Softening proceeded faster and the variation in firmness over time decreased rapidly for “Keitt” batches 5, 8, and 9, even with lower external ethylene levels applied than the recommended 100 μL L^−1^ (Kader, 1997). This would indicate that applying ethylene is an excellent tool to speed up ripening and decrease firmness variation. However, this is not the case for “Keitt” batches 6 and 7, that show only a minimal effect on softening when ethylene is applied. From a commercial viewpoint, the extra handling required moving the batches to sealed ripening rooms and applying ethylene does not result in a reliable softening effect and is therefore often abandoned.

Perhaps the most likely reason for the variation in ethylene induced softening is that mangoes, after long cool reefer transport, become less sensitive to ethylene due to prolonged chilling. This might be the case as batches with the shortest transport time, the batches from Israel (batch 5) and Spain (batch 8) show the highest softening after ethylene application compared to control (Table 1). It is, however, unclear why the Israeli batch showed higher ethylene induced softening as cooled transport time was longer than for the Spanish batch. From a modeling point of view, chilling might modify the ethylene factor. This could be investigated by estimating EF for sub-batches of a freshly harvested mango batch that is pre-treated with a varying duration of cold storage for each sub-batch.

### Multiple sources of firmness variation limit predictive power of the firmness model

Softening behavior of individual mangoes within a batch is sometimes faster or slower than the average behavior. This leads to simulated firmness lines depicting softening for a number of individual mangoes within a batch that crosses that of other mangoes (Figures 5–7, 10). This might indicate that the two individual sources of variation, the initial firmness and EF are independent. This is also indicated by the low correlation coefficient between these two stochastic variables varying between −0.3 and 0.3 for all batches (data not shown). As the stone is one of major ethylene producing parts of the mango (Reddy and Srivastava, 1999), it might be hypothesized that the stone size varies per batch. An additional complicating factor is the large variation in *F*_*fix*_ values for batches of the same cultivar (Table 1). The source of this variation is currently unknown. So, it seems that, next to the initial firmness and EF, also the final firmness varying per batch is a source of firmness variation that needs to be accounted for when considering applying the proposed firmness model for RTE predictions. In principle, describing multiple sources of variation in a stochastic model is not problematic (Hertog et al., 2007b; Jordan and Loeffen, 2013). For instance, when firmness data were ranked and when the three sources of variation (initial firmness, ethylene level and final firmness) are assumed to be normally distributed, then quantile regression (Jordan and Loeffen, 2013; Tijskens et al., 2017) worked well to describe the variation over time for batches shown in Table 1. However, for this approach to become predictive, we need information on two out of three sources of variation.

## Conclusions

With this model an all-encompassing view on mango firmness is presented. The proposed model is able to describe mango softening of prior cold transported mangoes accurately, including effects of the sourcing area, storage temperature, and ethylene application. One of the key assumptions of the model is that ethylene levels are constant over time. This was confirmed by ethylene measurements over time in the seed cavity. Ethylene application often enhances softening for batches that have a short-cooled transport time but this effect is typically smaller for reefer transported batches. The effect of ethylene application on firmness behavior could very well be described by estimating the ethylene factor, being a reflection of the internal ethylene level and the ethylene sensitivity. Temperature has a large effect on the ethylene factor. Hurdles that needs to be clarified in further research are the nature of the ethylene factor and the final firmness level that varies substantially per batch.

## Author contributions

SF, YW, and NK contributed to this work in data acquisition. RS and LT performed the analysis and interpretation of data. RS drafted the manuscript. EW, JV, NK, and LT contributed to data interpretation and discussion. EW, JV, NK, and LT critically revised and approved the manuscript.

### Conflict of interest statement

The authors declare that the research was conducted in the absence of any commercial or financial relationships that could be construed as a potential conflict of interest.
